# Effects on Iron Metabolism and System ${X_{c}}^{-}$ /GPX4 Pathway from Hydroquinone Suggest Ferroptosis of Jurkat Cells

**DOI:** 10.3390/toxics12090644

**Published:** 2024-08-31

**Authors:** Nana Liu, Ge Liu, Qiang Li, Yipeng Hu, Hong Wang

**Affiliations:** 1 Department of Occupational and Environmental Health, School of Public Health, Wuhan University, Wuhan 430071, Chinalqlq328328@163.com (Q.L.); huyipeng666@163.com (Y.H.); 2 Wuhan Center for Disease Control & Prevention, Wuhan 430000, China; liuqiuge876@163.com

**Keywords:** hydroquinone, ferroptosis, iron metabolism, System X_*c*_^−^, GPX4, leukaemia

## Abstract

Prolonged exposure to hydroquinone (HQ), a metabolite of benzene, can cause severe haematologic disorders in humans. However, the mechanism is still unclear. In the present study, we investigated whether HQ can induce haematological diseases through ferroptosis, which is another form of cell death apart from apoptosis. The results showed that HQ inhibited the viability of Jurkat cells in a dose-dependent and time-dependent manner. The half inhibitory concentrations (IC50s) of HQ-treated Jurkat cells for 12 h, 24 h and 48 h were 107.16 μmol/L, 33.29 μmol/L, and 14.78 μmol/L. The exposure of Jurkat cells to HQ increased intracellular ${\mathrm{Fe}}^{2+}$, malondialdehyde (MDA) and lipid reactive oxygen species (ROS) levels and down-regulated glutathione (GSH) levels. We used erastin-treated cells as a positive control and cells treated with HQ combined with deferoxamine mesylate (DFO) and ferrostain-1 (Fer-1)-treated cells as the negative controls. DFO and Fer-1 partially restored the degradation of cell viability and GSH content and the accumulation of ${\mathrm{Fe}}^{2+}$, MDA and lipid ROS caused by HQ. In addition, we found that cellular mitochondria in the HQ-treated group showed a decrease in volume, an increase in the density of the bilayer membrane and a decrease or disappearance of mitochondrial cristae. Changes in the erastin-treated group were similar to those in the HQ-treated group. We inferred that HQ induces ferroptosis in Jurkat cells. Subsequently, we found that HQ up-regulated the levels of transferrin receptor 1 (TFRC) mRNA and protein expression and down-regulated FTH1, SLC7A11 and synthetic substrate of antioxidant enzyme 4 (GPX4) mRNA levels and protein expression levels. However, the exposure of Jurkat cells to HQ with DFO and Fer-1 alleviated these changes. Notably, the activation of TFRC and the inhibition of FTH1 and System ${X_{c}}^{-}$ (cystine–glutamate reverse transporter protein) /GPX4 were associated with HQ-induced ferroptosis. These results provide novel insights into how HQ exacerbates haematopoietic cytotoxicity and provide potential targets for the prevention of HQ-induced diseases.

## 1. Introduction

In recent years, environmental pollutants have seriously jeopardised human health through indirect and direct exposure. Chronic human exposure to benzene can increase the risk of leukaemia [1,2,3], and the metabolites of benzene, such as hydroquinone (HQ), are key factors for various types of leukaemia [4,5,6]. However, the mechanism by which HQ causes leukaemia has not yet been fully elucidated. Consequently, exploring the mechanisms underlying HQ-induced leukaemia is essential.

Dixon et al. discovered a unique iron-dependent non-apoptotic form of cell death, which they subsequently named ferroptosis. It is different from apoptosis, necrosis and autophagy in morphology, biochemistry and genetics [7,8]. Since the discovery of ferroptosis, its role and regulatory mechanisms in the haematological system have been explored [9,10]. The continuous activity of haematopoietic stem cells (HSCs) is critical to the production of blood haematopoiesis [11,12], but studies have shown that HSCs are vulnerable to ferroptosis [13]. Thus, whether the mechanism underlying the toxic effects of HQ—an important factor in inducing haematologic toxicity—is related to ferroptosis requires further research.

Oxidative stress is one of the mechanisms underlying HQ-induced haematological diseases [14,15]. However, the process that regulates HQ-induced reactive oxygen species (ROS) production remains unknown. Studies have shown that iron metabolism is also related to oxidative stress [16,17]. When an excessive level of iron is present in a cell, a Fenton reaction occurs and directly generates ROS that damage the cell membrane [7]. In addition, System ${X_{c}}^{-}$, a cystine–glutamate reverse transporter protein, is a crucial intracellular antioxidant system, which is closely related to the synthesis of the antioxidant glutathione (GSH) [18]. GSH is an endogenous substrate of antioxidant enzyme 4 (GPX4), which maintains intracellular redox homeostasis by inhibiting lipid peroxidation [19]. Notably, iron metabolism and the System ${X_{c}}^{-}$/GPX4 pathway are closely associated with ferroptosis [8,20,21]. Given the above mentioned, we speculated that HQ can induce haematologic diseases through ferroptosis. This study provides novel insights into the mechanism of benzene and HQ in the development of leukaemia.

In this study, we investigated the possibility that HQ induces ferroptosis in cells and leads to haematological diseases by triggering the accumulation of cellular ROS. Furthermore, we studied whether iron metabolism and the System ${X_{c}}^{-}$/GPX4 pathway are involved in the regulation of these processes.

## 2. Methods

### 2.1. Cell Culture and Reagents

Jurkat cells, belonging to a T-cell acute lymphoblastic leukaemia cell line, have the properties of bone marrow haematopoietic stem cells (HSCs). The molecular mechanisms of haematopoietic toxicity caused by benzene and its metabolites have been extensively studied in various leukaemia cell lines. Cells were cultured in RPMI-1640 (Biological Industries, ISR) containing 10% foetal bovine serum (Biological Industries, ISR), 100 U/mL penicillin (Hyclone, Logan, UT, USA) and 100 U/mL streptomycin (Hyclone, USA), and then incubated in a humid atmosphere containing 5% CO_2_ at 37 °C. Logarithmic growth-phase cells were collected for subsequent experiments. HQ was purchased from MACKLIN, Taipei, Taiwan, China, and Erastin, ferrostain-1 (Fer-1) and deferoxamine mesylate (DFO) were purchased from MedChemExpress, MCE, Monmouth Junction, NJ, USA.

### 2.2. Cell Viability Assays

Jurkat cells were inoculated into 96-well plates at 2 × 10^4^ cells per well and treated with different concentrations of HQ solutions, which were made with dd-H_2_O and inhibitors for 24 h. Then, 0.1% cell counting kit-8 (CCK-8) solution (Dojindo, Kumamoto, Japan) was added to each well, and the cells were incubated for 1–2 h at 37 °C. After that, the OD value of each well at 450 nm was measured on an enzyme-labelling instrument (Biotek, Shoreline, WA, USA).

### 2.3. Determination of Intracellular $Fe^{2+}$ Content

Cells at the end of incubation were collected and washed twice with HBSS (Hank’s Balanced Salt Solution, 0.1 M, pH 7.4) buffer, resuspended in 1 μmol/L FerroOrange working solution and inoculated in a six-well plate. Incubation was continued for 30 min, and then the cells were observed with a fluorescence microscope (OLYMPUS, IX3-SVR, Tokyo, Japan) under a green laser (Ex: 532 nm).

### 2.4. Detection of Intracellular GSH and Malondialdehyde (MDA) Levels

Absorbance was measured at 405 and 532 nm with GSH and malondialdehyde (MDA) assay kits (both from Nanjing Jiancheng Bioengineering Institute, Nanjing, China) according to the manufacturers’ instructions.

### 2.5. Detection of Intracellular Lipid ROS Content

The cells were collected through centrifugation, and the supernatant was discarded. The cells were resuspended in a prepared diluted BODIPY 581/591 C11 working solution (BODIPY 581/591 C11 storage solution/complete medium = 1:1000) and incubated for 30 min in the dark. After incubation, the cells were washed with HBSS buffer once or twice and finally resuspended in 500 μL of HBSS. The intracellular lipid ROS content was detected by flow cytometry (CytoFLEX S, USA) FITC Filter.

### 2.6. Observation of Mitochondrial Ultrastructure

The cells were collected through centrifugation at the end of processing, and the supernatant was discarded. Then, 1 mL of 2.5% glutaraldehyde fixative was added to the cell precipitate and fixed at 4 °C. Thereafter, cells were rinsed with 0.1 M phosphate buffer three times, centrifugation was performed, and the precipitate was encapsulated in agarose. The sample was fixed with 1% osmium acid (OsO4) at room temperature away from light for 2 h, and the above rinsing steps were performed three times. The plates were dehydrated with different concentrations of ethanol and osmotically embedded. The embedded plates were polymerised in an oven at 60 °C for 48 h, and resin blocks were removed and set aside. The samples were then cut into ultrathin sections with an ultrathin slicer (Leica, GER, Thuringia, Germany), and the sections were stained sequentially with 1% uranyl acetate–saturated alcohol and 1% lead citrate solutions. The ultrastructure of cellular mitochondria was observed by transmission electron microscopy (Hitachi, Ibaraki, Japan).

### 2.7. Total RNA Extraction, Reverse Transcription and Quantitative Real-Time PCR

Total RNA was extracted from the cells according to the instructions of Trizol (Beyotime, China), and the RNA concentration was measured and adjusted to the same concentration in each group. Total RNA (1 μg) was reverse-transcribed to a volume of 20 μL with a T100TM thermal cycler (BIO RAD, USA). Human transferrin receptor 1 (TFRC), FTH1, SLC7A11 and GPX4 genes were designed by combining the full sequences and primer information published by GenBank and Primer Bank, and the specificity was verified by Blast. A qTOWER^3^ real-time fluorescence quantitative PCR instrument (Analyik Jena, GER, Jena, Germany) was used for real-time PCR in a 20 μL reaction system. The relative mRNA expression level was calculated using the $2^{-△△\mathrm{CT}}$ method in Excel Microsoft 2023.

### 2.8. Protein Extraction and Western Blot Analysis

Cells were collected and centrifuged, and the supernatant discarded and washed once with pre-cooled PBS (0.1 M, pH 7.4). A lysis solution (RIPA/protease inhibitor = 100:1, Beyotime, China) was added, and the cells were vortexed every 5 min. This procedure was performed six times, and lysis was conducted on ice for 10 min. Centrifugation was performed at 12,000 rpm for 20 min, and the supernatant was collected. A 5× Sodium dodecyl sulfate (SDS) sampling buffer was added, and denaturation was conducted at 98 °C for 10 min. Then, 30–40 µg of total protein was collected for sampling. GAPDH protein was used as an internal reference, and the proteins were separated by 10% and 12% SDS-PAGE and transferred to polyvinylidene difluoride (PVDF) membranes. The PVDF membrane was cut according to the instructions of the protein marker, blocked with 5% skimmed milk powder for 2 h and then incubated with TFRC, FTH1, SLC7A11, GPX4 and GAPDH primary antibodies at 4 °C overnight. On the next day, the membrane was washed three times with TBST (Tris-Buffered Saline buffer/Tween 20 = 1000:1) buffer, and horseradish enzyme–labelled secondary antibody was added, and the membrane was incubated at room temperature for 2 h. The membrane was washed three times, and the ECL reagent method was used for luminescence colour development. Image J 1.54 software was used to analyse the grey value of each protein, and the final results were the target protein/β-actin ratios. TFRC, SLC7A11, GPX4, GAPDH primary antibody and secondary antibody were obtained from Proteintech, Wuhan, China; FTH1 primary antibody was obtained from HUABIO, China; and ECL reagent was obtained from Beyotime, China.

### 2.9. Statistical Analysis

SPSS22.0 software was used for the statistical analysis. A one-way analysis of variance was performed to compare differences among multiple groups. Dunnett’s *t*-test was utilised to analyse differences between the experimental and control groups. Differences were statistically significant when *p* < 0.05.

## 3. Results

### 3.1. HQ Has a Significant Toxic Effect on Jurkat Cells

**Figure 1:**
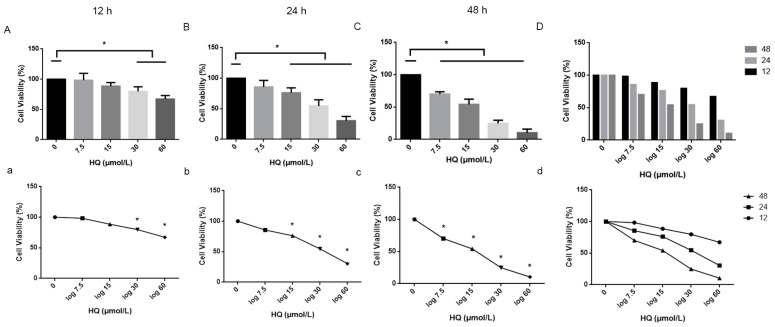
Changes in the viability of Jurkat cells treated with different concentrations of HQ for different durations. (1) (**A**–**D**, **a**–**d**): Subsequently, Jurkat cells were treated with different concentrations of HQ for 12, 24 and 48 h. (2) Considering the time effect and the validity of the follow-up experiments, HQ at a concentration of 15 µmol/L was selected to be treated for 24 h for the follow-up experiments. (3) *: *p* < 0.05 compared to control group, *n* = 3 replicate independent experiments.

The toxic effect of HQ on Jurkat cells was assessed according to the effect of HQ on Jurkat cell viability with a CCK-8 kit. Jurkat cells were treated with 0, 7.5, 15, 30 and 60 μmol/L HQ for 12, 24 and 48 h. The proliferation of the Jurkat cells was significantly inhibited. Furthermore, the proliferation of the Jurkat cells decreased with an increasing HQ concentration and treatment time (Figure 1). In contrast to the control, HQ had a significant toxic effect on the Jurkat cells, and this toxic effect was accompanied by dose-dependent and time-dependent effects.

**Figure 2:**
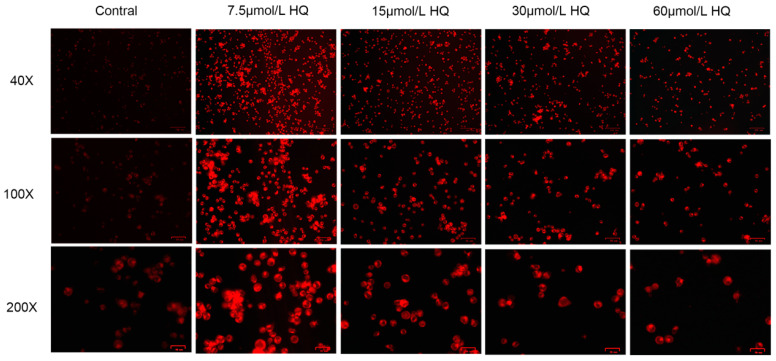
Changes in the concentration of ${\mathrm{Fe}}^{2+}$ in HQ-treated group. The 40×, 100× and 200× magnifications are 100, 50 and 25 µm, respectively.

The previous results showed that HQ exerts toxic effects on Jurkat cells, but the mechanism underlying HQ toxicology is still unclear. Studies have shown that iron metabolism is related to oxidative stress. To determine whether the content of iron contributes to HQ toxicology, we quantified ${\mathrm{Fe}}^{2+}$ in the HQ-treated group with a FerroOrange fluorescence probe. A comparison between the HQ and control groups indicated that HQ can increase the intracellular concentration of ${\mathrm{Fe}}^{2+}$ (Figure 2). FerroOrange is a novel fluorescence probe that is sensitive to ${\mathrm{Fe}}^{2+}$ in live cells. The survival rate of Jurkat cells decreases with an increasing HQ concentration (Figure 1). Although a considerable increase in the levels of ${\mathrm{Fe}}^{2+}$ occurred in the HQ-treated group at all concentrations, cell viability decreased with an increasing HQ concentration (Figure 2). This effect led to a decrease in the intensity of FerroOrange fluorescence in the 30 and 60 μmol/L HQ-treated groups.

**Figure 3:**
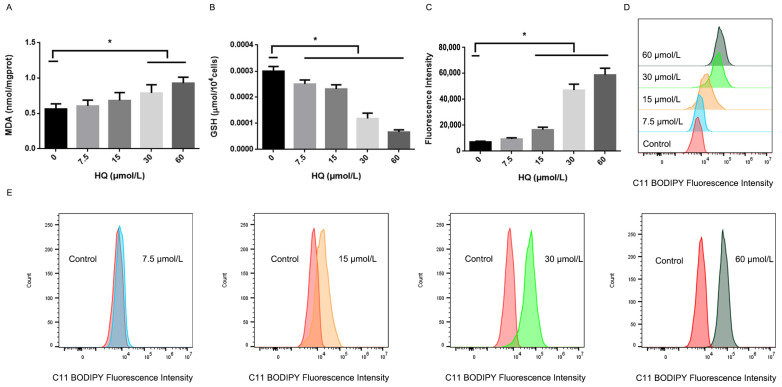
Changes in the cellular antioxidant system in the HQ-treated group. (1) (**A**) The levels of MDA in the Jurkat cells. (2) (**B**): The levels of GSH in the Jurkat cells. (3) (**C**–**E**): The levels of lipid ROS in the Jurkat cells. (4) *: *p* < 0.05 compared with the control group, *n* = 3 replicate independent experiments.

In addition to the detection of intracellular ${\mathrm{Fe}}^{2+}$, we examined the redox homeostasis of the cells. The content of MDA, one of the end products of lipid peroxidation, and lipid ROS can reflect the degree of the redox homeostasis inside cells [7]. The levels of MDA in the 30 and 60 μmol/L HQ-treated groups were significantly higher than those in the control group (Figure 3A). The GSH content can reflect the ability of cells to remove ROS to a certain extent [22]. Figure 3B shows that the intracellular GSH content of the Jurkat cells gradually decreased with an increasing HQ concentration (Figure 3C–E). As the HQ concentration increased, the ROS peak curve (Figure 3D) shifted to the right, the BODIPY 581/591 C11 fluorescence intensity gradually increased and intracellular lipid ROS content gradually increased. A BODIPY 581/591 C11 probe is a fluorescent probe for detecting lipid peroxidation. These results demonstrated that HQ leads to the inhibition of the cellular antioxidant system, further demonstrating that ferroptosis occurs in cells.

### 3.2. HQ Induces Ferroptosis in Jurkat Cells

**Figure 4:**
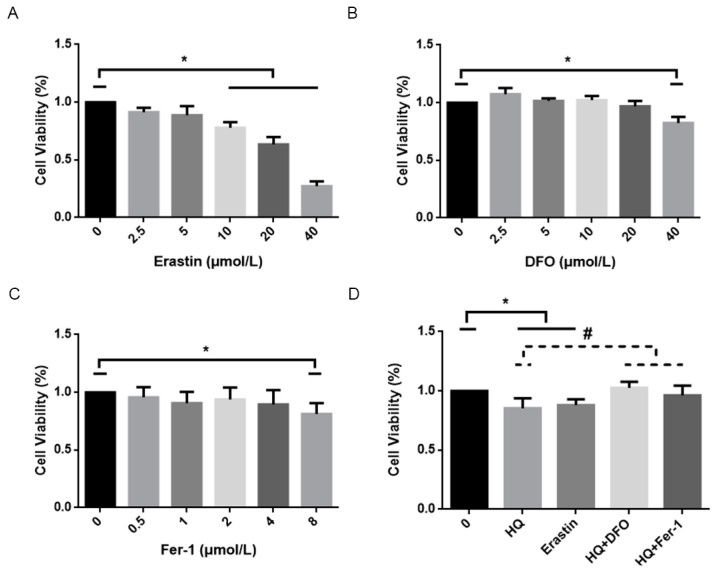
Changes in the viability of Jurkat cells treated with 15 µmol/L of HQ and other inducers (erastin) and inhibiters of ferroptosis (DFO and Fer-1) for 24 h. (**A**): The viability of Jurkat cells treated with erastin. (**B**): The viability of Jurkat cells treated with DFO. (**C**): The viability of Jurkat cells treated with Fer-1. (**D**) The viability of Jurkat cells treated with HQ, erastin and HQ with DFO and Fer-1. (1) To avoid the toxic effect of HQ on Jurkat cells, we chose the concentration at which they did not affect the activity of Jurkat cells. The concentrations of erastin, DFO and Fer-1 used in subsequent experiments were 10, 20 and 4 μmol/L, respectively. (3) *: *p* < 0.05 compared to control group, *n* = 3 replicate independent experiments; #: *p* < 0.05 compared to HQ-treated group, *n* = 3 replicate independent experiments.

The above results suggest that HQ may induce cytotoxicity through ferroptosis. The cytotoxic effect of HQ on Jurkat cells was further explored by using erastin-treated cells as the positive control and HQ combined with DFO and Fer-1-treated cells as the negative controls. Erastin is a known ferroptosis inducer [23], and DFO is an iron chelator that combines with intracellular iron ions and then reduces intracellular iron content and inhibits cellular ferroptosis. Fer-1, unlike DFO, inhibits ferroptosis by inhibiting the formation of lipid peroxides. The cell viability in the HQ-treated and erastin-treated groups was lower than that in the blank control group (Figure 4D). Compared with the HQ-treated group, the DFO, Fer-1 and HQ combined treatment groups exhibited higher cell survival rates than the HQ-treated group, suggesting that DFO and Fer-1 can partially alleviate cellular damage caused by HQ.

**Figure 5:**
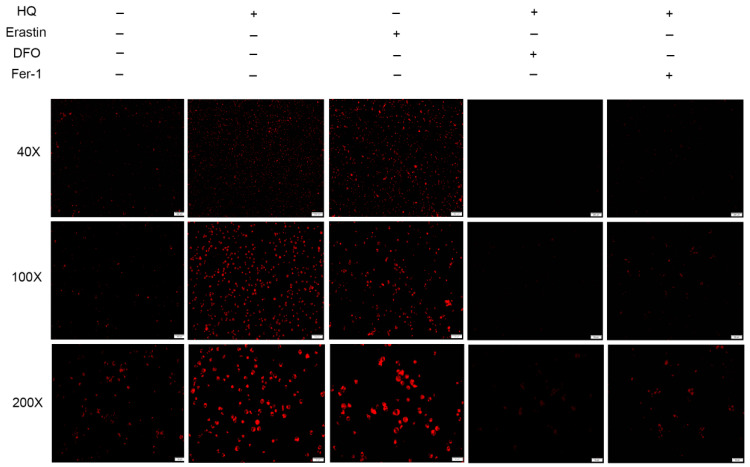
Changes in the concentration of ${\mathrm{Fe}}^{2+}$ of each treatment group. The 40×, 100× and 200× magnifications are 200, 100 and 50 µm, respectively.

Ferroptosis is a form of non-apoptosis cell death, and it depends on iron. Heat shock protein ꞵ-1 decreases intracellular iron concentrations and inhibits ferroptosis [24]. To demonstrate the regulatory effect of HQ on ferroptosis in Jurkat cells, we compared the cellular ${\mathrm{Fe}}^{2+}$ content of the HQ- and erastin-treated groups and the group treated with DFO and Fer-1 combined with HQ. The HQ-treated and erastin-treated groups showed increased intracellular ${\mathrm{Fe}}^{2+}$ levels compared with the blank control group. Compared with the HQ-treated group (Figure 5), the group treated with HQ combined with DFO and the Fer-1-treated group inhibited the ${\mathrm{Fe}}^{2+}$ accumulation caused by HQ. This result suggested that DFO and Fer-1 can alleviate the cytotoxic effects of HQ by regulating intracellular ${\mathrm{Fe}}^{2+}$ content.

**Figure 6:**
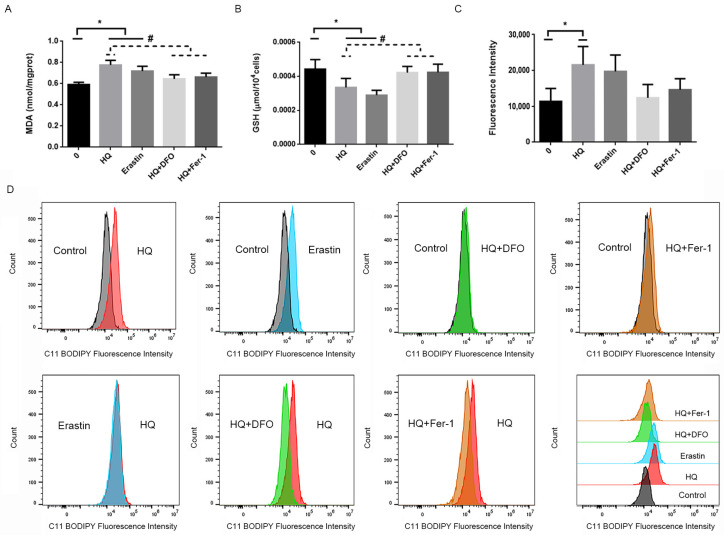
Changes in the cellular antioxidant system of each treatment group. (1) (**A**) The levels of MDA in the Jurkat cells. (2) (**B**): The levels of GSH in the Jurkat cells. (3) (**C**,**D**): The levels of lip ROS in the Jurkat cells. (4) *: *p* < 0.05 compared with the control group, *n* = 3 replicate independent experiments; #: *p* < 0.05 compared with the HQ-treated group, *n* = 3 replicate independent experiments.

An increase in ${\mathrm{Fe}}^{2+}$ content is one of the biochemical features of ferroptosis [25], in addition to the accumulation of lipid peroxidation, such as lipid ROS. To evaluate intracellular ferroptosis, we not only detected the ${\mathrm{Fe}}^{2+}$ content but also the function of the cellular antioxidant system. The results showed that the intracellular MDA (Figure 6A) and lipid ROS content were higher in the HQ- and erastin-treated cells than in the blank control group (Figure 6C,D). In contrast, GSH content was lower than that in the blank control (Figure 6B). The intracellular MDA and lipid ROS content of the group treated with HQ combined with DFO and the Fer-1 treatment group were lower than those of the HQ-treated group, but the content in all groups was not significantly different from that of the blank control group (Figure 6A,C,D). Moreover, the intracellular GSH content was higher than that of the HQ-treated group. The results showed that HQ and erastin induced lipid peroxidation in the Jurkat cells, whereas DFO and Fer-1 partially restored the antioxidant function of the cells.

**Figure 7:**
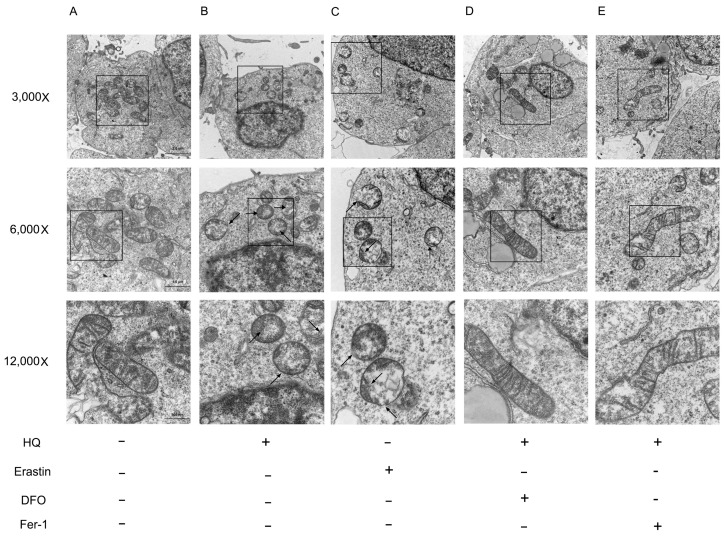
Changes in mitochondria in each treatment group. (**A**–**E**): Changes in mitochondria in control, HQ, Erastin, HQ+DFO and HQ+Fer-1treatment group. (1) The 3000×, 6000× and 12,000× scales are 2.0 µm, 1.0 µm and 500 nm, respectively. (2) Black arrows point to areas where mitochondrial cristae are reduced or absent, the density of the bilayer is increased, and the outer membrane is ruptured.

To further determine the mode of death, we observed mitochondrial changes under different treatment conditions using transmission electron microscopy. The typical morphological changes in ferroptosis were a decreased volume of mitochondria, decreased or disappeared cristae and increased density of the bilayer membrane [7]. As shown in Figure 7, the mitochondria of cells in the HQ- and erastin-treated groups showed changes consistent with ferroptosis, whereas the mitochondria of cells in the group treated with HQ combined with DFO and the Fer-1-treated group were consistent with those of the blank control group. In conclusion, we determined that ferroptosis plays a key role in HQ-induced cellular injury.

### 3.3. Iron Metabolism Pathway and System ${X_{c}}^{-}$/GPX4 Pathway Involved in HQ-Induced Ferroptosis

**Figure 8:**
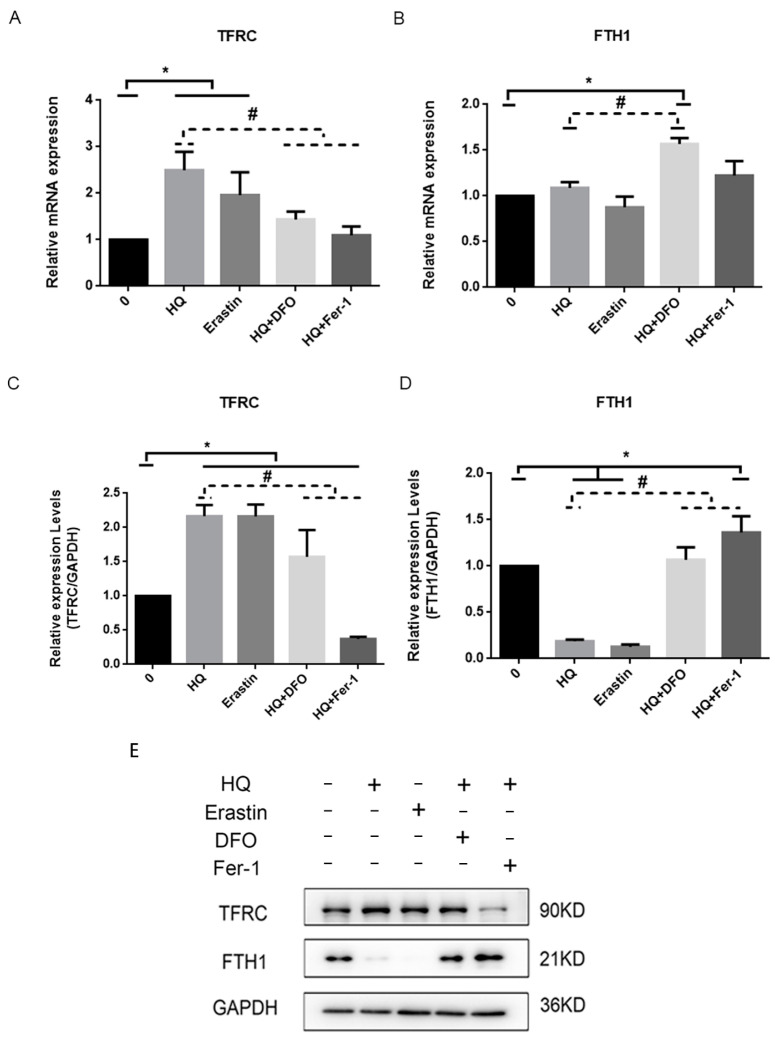
(**A**,**B**): Expression levels of TFRC and FTH1 mRNA in each treatment group. (**C**–**E**) Expression levels of TFRC and FTH1 proteins in each treatment group. (1) *: *p* < 0.05 compared with control group, *n* = 3 replicate independent experiments; #: *p* < 0.05 compared with the HQ-treated group, *n* = 3 replicate independent experiments.

TFRC and ferritin are well known players in the homeostasis of intracellular iron [26]. TFRC internalises iron bound to transferrin. FTH1 is a subunit of ferritin that stores iron and regulates intracellular ${\mathrm{Fe}}^{2+}$, thereby affecting cellular sensitivity to ferroptosis [27]. Thus, intracellular ${\mathrm{Fe}}^{2+}$ content is closely related to HQ-induced ferroptosis. We investigated changes in TFRC/FTH1 mRNA and protein expression levels in different treatment groups (Figure 8). TFRC mRNA and protein expression levels were higher in the HQ- and erastin-treated groups than in the blank control group and lower in the group treated with HQ combined with DFO and the Fer-1-treated group than in the HQ-treated group (Figure 8A,C). The FTH1 protein expression in the group treated with HQ combined with DFO and the Fer-1-treated group was higher than that in the HQ-treated group, and the protein expression in the HQ- and erastin-treated groups was lower than that in the blank control (Figure 8D). These results suggested that HQ affects intracellular ${\mathrm{Fe}}^{2+}$ content through the activation of TFRC and the inhibition of FTH1 expression, which in turn mediate the sensitivity of Jurkat cells to ferroptosis.

**Figure 9:**
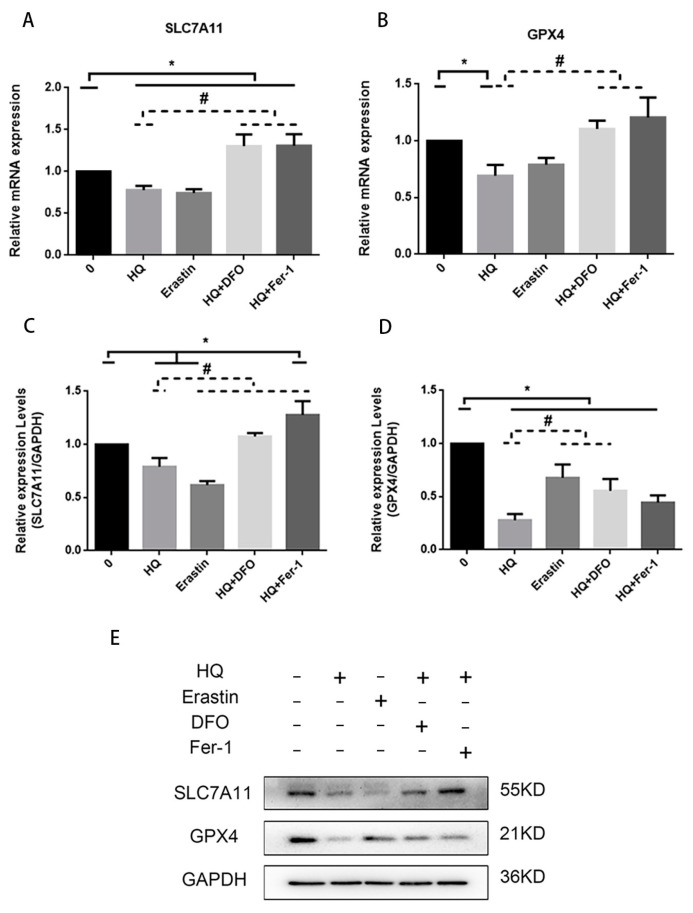
(**A**,**B**) Expression levels of SLC7A11 and GPX4 mRNA in Jurkat cells under each treatment. (**C**–**E**) Expression levels of SLC7A11 and GPX4 proteins in Jurkat cells under each treatment. *: *p* < 0.05 compared with the control group, *n* = 3 replicate independent experiments; #: *p* < 0.05 compared with the HQ-treated group, *n* = 3 replicate independent experiments.

In addition to iron metabolism, we investigated whether System ${X_{c}}^{-}$/GPX4 is involved in HQ-induced ferroptosis. SLC7A11 is one of the components of the transmembrane protein System ${X_{c}}^{-}$, and we examined the levels of SLC7A11 and GPX4 mRNA and protein expression in the cells of different treatment groups. The SLC7A11 mRNA and protein expression levels in the HQ- and erastin-treated groups were lower than those in the blank group, and the down-regulation of SLC7A11 mRNA and protein caused by HQ was partially alleviated by DFO and Fer-1 (Figure 9A,C). Changes in GPX4 mRNA and protein expression levels were similar to those in the levels of System ${X_{c}}^{-}$, and the HQ-treated group exhibited down-regulated GPX4 mRNA and protein expression levels, which were partially inhibited by DFO and Fer-1 (Figure 9B,D). This result suggested that the System ${X_{c}}^{-}$/ GPX4 signalling pathway is involved in HQ-induced ferroptosis in Jurkat cells.

## 4. Discussion

HQ is a metabolite of benzene, which is used in a variety of commercial and industrial processes and is a commonly used contaminant [3]. Although HQ can induce cytotoxicity by inhibiting DNA synthesis [28], inducing apoptosis [29] and controlling the cell cycle [30], little is known about the link between HQ and ferroptosis and its specific regulatory mechanisms. In the present work, we found that HQ can exert cytotoxic effects through ferroptosis, which is accompanied by an increase in intracellular free ${\mathrm{Fe}}^{2+}$ and a dysregulation of redox system homeostasis. In addition, we observed that the activation of TFRC and inhibition of FTH1 and System ${X_{c}}^{-}$/GPX4 may be associated with HQ-induced ferroptosis.

Some studies have shown that HQ-induced ROS production is associated with the cytotoxicity of HQ. In this study, we firstly treated Jurkat cells with different concentrations of HQ. The result showed that the proliferation of Jurkat cells was inhibited in a dose-dependent and time-dependent manner. In addition to that, Jurkat cells are generated by the abnormal growth and differentiation of bone marrow haematopoietic stem cells and polyclonal malignant proliferation, and they have the characteristics of bone marrow haematopoietic stem cells. Due to the multidirectional differentiation of haematopoietic stem cells, they are generally not cultured in vitro. Therefore, in addition to haematopoietic stem cells purified from human or mouse bone marrow, various leukaemia cell lines have been used in domestic and international studies to study the haematological toxicity of benzene and its metabolites and the related molecular mechanisms [4,31,32,33,34]. The toxic effect of HQ on cells was confirmed. Subsequently, we found that HQ increased intracellular MDA and lipid ROS content and down-regulated GSH content. Interestingly, this result is consistent not only with previous studies [14,15,33] but also with one of the biochemical features of ferroptosis [35,36]. Ferroptosis, which has been a major research topic since its discovery by Dixon et al., is an iron-dependent non-apoptotic form of cell death. It is associated with two major biochemical features: iron accumulation and lipid peroxidation. Studies have shown that as the intracellular level of ${\mathrm{Fe}}^{2+}$ increases, so does the susceptibility of cells to ferroptosis. [7,37]. Notably, our results showed that HQ not only caused the accumulation of intracellular lipid peroxides but also up-regulated the intracellular ${\mathrm{Fe}}^{2+}$ content, consistent with the characteristics of ferroptosis. Therefore, we hypothesised that HQ induces cytotoxicity through ferroptosis.

To verify the above idea, we used erastin, a known inducer of ferroptosis, as a positive control and DFO, an iron chelator, and Fer-1, a ferroptosis inhibitor, as negative controls. Then, we measured the corresponding ferroptosis indexes. The results showed that DFO and Fer-1 partially inhibited the decrease in cell viability caused by HQ. In addition, they partially mitigated the HQ-induced accumulation of MDA, lipid ROS and intracellular ${\mathrm{Fe}}^{2+}$ and the reduction in GSH. It is suggested that HQ may induce ferroptosis in Jurkat cells. Ferroptosis is mainly characterised by a decrease in mitochondrial volume, an increase in the density of the bilayer membrane and a decrease or disappearance of the mitochondrial cristae [7]. Our results showed that the mitochondria of cells in the HQ- and erastin-treated groups showed the above-mentioned features of ferroptosis. However, the cells in the group treated with HQ combined DFO and the Fer-1-treated group did not. This finding further validated that HQ induces ferroptosis in cells.

Excess intracellular ${\mathrm{Fe}}^{2+}$ contributes to the cellular sensitisation to ferroptosis, and thus iron metabolism plays a crucial role in this process [38]. Genes or proteins involved in iron metabolism are involved in the regulation of cellular sensitivity to ferroptosis [39,40]. TFRC can recognise transferrin and deliver ${\mathrm{Fe}}^{3+}$ bound to transferrin into a cell. Then, ${\mathrm{Fe}}^{3+}$ is reduced to ${\mathrm{Fe}}^{2+}$ and subsequently stored in an unstable iron pool and ferritin. Pei et al. found that the inhibition of TFRC expression prevents lung fibrosis by interrupting ferroptosis in fibroblasts [26]. FTH1 is a subunit of ferritin, which binds and stores intracellular free iron to regulate intracellular iron content [34]. The down-regulation of FTH1 promotes ferroptosis in bladder cancer cells [27]. Our results showed that the HQ-treated group up-regulated the levels of TFRC mRNA and protein expression and down-regulated the protein expression level of FTH1. Furthermore, DFO and Fer-1 can alleviate the above changes, suggesting that the activation of TFRC and the inhibition of FTH1 are involved in HQ-induced ferroptosis. However, the regulation of iron homeostasis is a very complex process, and studies have shown that iron homeostasis is mainly dependent on the regulation of iron regulatory protein–iron responsive element (IRP-IRE). The IRP-IRE system is iron homeostasis’s central regulator and critical component [41]. This study indicates that IRP1/iron-responsive element-binding protein 2 (IRP1/2) binds to mRNAs located in the FTL, FTH1 and SLC40A1 mRNAs and inhibits their translation by preventing small ribosomal subunits from binding to the mRNAs [42]. In contrast, iron-responsive element (IRP) interacts with TFRC mRNA to protect transcripts from endonuclease attack [43]. Thus, IRP-IRE binding reduces iron storage and export and promotes iron uptake, thereby increasing cellular iron bioavailability. However, whether the IRP-IRE system plays a regulatory role in HQ-induced ferroptosis in Jurkat cells requires further study.

In addition, System ${X_{c}}^{-}$ is involved in the regulation of ferroptosis to a certain extent [8,39,44]. Cystine and glutamate are exchanged in and out of cells by System ${X_{c}}^{-}$ in a ratio of 1:1. Subsequently, cystine is reduced to cysteine in the cells and promotes GSH synthesis [7]. Antioxidant GSH is an endogenous substrate for GPX4, which is a crucial antioxidant enzyme in the human body. GPX4 converts GSH into oxidised glutathione and reduces cytotoxic lipid peroxides (PL-OOH) to the corresponding alcohols (PL-OH), thereby preventing the synthesis of lipid peroxides [22]. The inhibition of System ${X_{c}}^{-}$-leads to a decrease in GSH synthesis and down-regulation of GPX4 expression, resulting in the weakening of intracellular antioxidant effects and the accumulation of lipid peroxides, which in turn increases the susceptibility of cells to ferroptosis [45]. In this study, we showed that HQ and erastin down-regulated SLC7A11 and GPX4 mRNA and protein expression levels, and DFO and Fer-1 partially alleviated the down-regulation caused by HQ. SLC7A11 is a component of the transmembrane protein System ${X_{c}}^{-}$. Thus, HQ may induce ferroptosis by inhibiting the System ${X_{c}}^{-}$/GPX4 signalling pathway. Although the TFRC/FTH1 and System ${X_{c}}^{-}$/GPX4 signalling pathways may be involved in HQ-induced ferroptosis, the specific upstream and downstream mechanisms involved in the regulation of ferroptosis remain unclear. These processes will be explored in the future with an enhanced experimental design.

In conclusion, the present study demonstrated that HQ induces cellular ferroptosis by promoting the accumulation of ${\mathrm{Fe}}^{2+}$, lipid ROS and MDA and by down-regulating GSH level. In addition, we found that the activation of TFRC and inhibition of FTH1 and System ${X_{c}}^{-}$/GPX4 are associated with HQ-induced ferroptosis. These results provide novel insights into how HQ exacerbates haematopoietic cytotoxicity and provide potential targets for the prevention of HQ-induced diseases. However, numerous epidemiologic and animal studies are needed to determine the specific mechanism underlying HQ-induced ferroptosis.

## Data Availability

The original data presented in the study are included in the article; further inquiries can be directed to the corresponding author.
